# Pharmacological Profile, Bioactivities, and Safety of Turmeric Oil

**DOI:** 10.3390/molecules27165055

**Published:** 2022-08-09

**Authors:** Adriana Monserrath Orellana-Paucar, María Gabriela Machado-Orellana

**Affiliations:** 1 Nutrition and Dietetics School, Faculty of Medical Sciences, University of Cuenca, Cuenca 010204, Ecuador; 2 Pharmacology and Nutritional Sciences Interdisciplinary Research Group, Faculty of Medical Sciences, University of Cuenca, Cuenca 010204, Ecuador; 3 Medicine and Surgery School, Faculty of Medical Sciences, University of Cuenca, Cuenca 010204, Ecuador

**Keywords:** *Curcuma longa*, turmeric, curcuma oil, turmeric oil, pharmacological profile, pharmacological activity, safety, toxicity

## Abstract

The pharmacological attributes of turmeric have been extensively described and frequently related to the action of curcuminoids. However, there is also scientific evidence of the contribution of turmeric oil. Since the oil does not contain curcuminoids in its composition, it is crucial to better understand the therapeutic role of other constituents in turmeric. The present review discusses the pharmacokinetics of turmeric oil, pointing to the potential application of its active molecules as therapeutic compounds. In addition, the bioactivities of turmeric oil and its safety in preclinical and clinical studies were revised. This literature-based research intends to provide an updated overview to promote further research on turmeric oil and its constituents.

## 1. Introduction

*Curcuma longa* L. (syn. *Curcuma domestica*), commonly known as turmeric, is a perennial herb native to Asia. After curing, drying, and milling, turmeric rhizomes are usually employed as a dye, cosmetic, and food seasoning. In traditional medicine, turmeric is used for treating hepatic and gastrointestinal disorders, arthritis, rheumatism, skin diseases, fever, inflammation, amenorrhea, sepsis, and as an anthelmintic and laxative [1,2,3]. Some of these properties are supported by scientific evidence, and other novel activities have also been uncovered.

**Figure 1:**
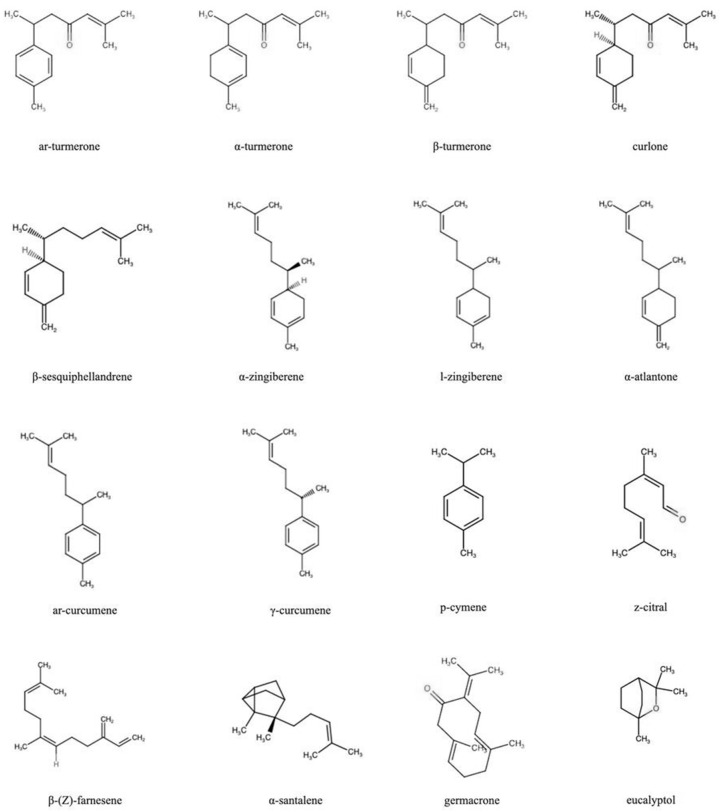
Chemical structure of bioactive turmeric oil constituents.

Most pharmacological activities of turmeric have been explained by the properties of curcumin, mainly because turmeric oil has not been as extensively studied as curcuminoids. Turmeric rhizome oil (TO) is responsible for this spice’s characteristic taste and smell. Dried rhizomes contain about 3–6% essential oil [4]. The oil is extracted from powdered turmeric rhizomes through steam distillation. The major TO constituents are sesquiterpenes: bisabolanes, guaianes, germacranes, caranes, elemanes, spironolactones, selinanes, santalanes, and caryophyllanes [5,6]. Ar-turmerone, α-turmerone, and β-turmerone are the principal bisabolane sesquiterpenes [6,7]. Other notable TO compounds with reported bioactivity are α-atlantone, ar-curcumene, γ-curcumene, curlone, p-cymene, z-citral, eucalyptol, β-(Z)-farnesene, germacrone, β-sesquiphellandrene, α-santalene, α-zingiberene, and l-zingiberene [1,5] (Figure 1).

## 2. Pharmacological Profile

The murine pharmacokinetics profile of TO (500 mg/kg; p.o.) revealed the oil capability to be absorbed after oral administration, the high bioavailability, and the extended residence time for ar-turmerone, α, β-turmerone, and curlone [8]. TO displayed a peak plasma level 2 h after administration. Plasma concentrations of ar-turmerone and α-,β-turmerone remained uniform (100–135 ng/mL) from 8 to 18 h. Ar-turmerone showed a bioavailability of 13%, α,β-turmerone 11%, and curlone 7%. The mean residence time was 13.2, 11.6, and 14 h, respectively [8]. The high bioavailability of ar-turmerone in mice brains suggests the feasibility of orally administering the oil (or its components) through prolonged dosage periods since the plasma concentration remains stable for a considerable time lapse [9].

Regarding excretion, ar-turmerone was detected intact in 24 h urine of healthy adult volunteers after dry curcuma extract consumption. In addition, two prostaglandin-D2 metabolites were identified. This finding aligns with the anti-inflammatory effect attributed to this plant [10].

Additionally, the role of TO as a bioavailability enhancer is of current interest. Amyloid-β peptide accumulation and increased β-secretase activity have been associated with Alzheimer’s dementia pathogenesis [11,12]. The lignans, sesamin and sesamolin, inhibit β-secretase. Nevertheless, both showed low bioavailability in murine models. TO acted as an efficient carrier, enhancing brain permeation of sesamin and sesamolin [13]. Likewise, the molecules responsible for the preventive effect of *Ginkgo biloba* in cases of dementia and Alzheimer’s disease are flavonoids and terpene lactones with a well-known poor brain bioavailability. This limitation was surpassed in mice by joint administration with TO and sesame extract [14]. When curcumin is co-administered with ar-turmerone, curcumin has a significant permeation increase in the Caco-2 cell monolayer [15]. The high brain bioavailability of TO is relevant not only for its role as an enhancer but also for its intrinsic therapeutic properties. For instance, the antitumor properties attributed to TO are interesting in neurology due to its capability to cross the blood–brain barrier [16].

## 3. Bioactivity

### 3.1. Antioxidant

Nitric oxide, superoxide, and peroxynitrite levels increase in response to infections or inflammation. The radical scavenging assay and the ferric reducing antioxidant power test displayed significant antioxidant activity for TO [17,18]. TO inhibited superoxide generation triggered by phorbol-12-myristate-13-acetate in mice after i.p. administration. Additionally, oral administration of TO for 30 days prompted a relevant increase in glutathione and antioxidant enzyme concentrations of superoxide dismutase and glutathione reductase in plasma (*p* < 0.001). In the liver, glutathione-S-transferase and superoxide dismutase concentrations were increased by TO’s influence (*p* < 0.01) [19]. TO decreased nitric oxide (NO) synthase expression and displayed immune-modulatory properties since it restricted neutrophil infiltration in the ischemic area of a murine cerebral focal ischemia model. Altogether, NO, peroxynitrite, and reactive oxygen neuronal concentration diminished and the number of apoptotic cells [20,21].

Moreover, carrageenan-induced inflammation is characterized by two phases: (a) rise in serotonin and histamine levels and (b) augment of proteases, prostaglandins, and superoxide concentrations [22]. The TO response in this model suggests a powerful influence as an antioxidant and critical modulator of inflammation mediators. In addition, it has been suggested that the capability of TO to scavenge free radicals and activate antioxidant enzymes may be related to its antimutagenic action [21].

### 3.2. Anti-Inflammatory

The models of acute inflammation stimulated by carrageenan and dextran and the chronic model induced by formalin were used to assess the anti-inflammatory properties of TO following i.p. administration in mice. In all cases, the oil reduced paw thickness. In the chronic model, the effect observed with TO was comparable to that obtained with diclofenac [19]. Accordingly, TO administration in rats significantly decreased paw edema in the carrageenan-induced inflammation model (76%) compared to aspirin. This reduction was only 62% when TO was administered with fish oil [23]. Thus, TO sesquiterpenes seems to act as antagonists or inhibitors of EPA and DHA action.

Furthermore, when employed as a pre- and post-treatment for myocardial ischemia/reperfusion (MI/RP), endothelial cell-mediated inflammation was lessened by TO in rats. TO reduced the ischemic area, adhesion of inflammatory cells to endothelial cells, and expression of pro-inflammatory genes and adhesion factors such as E-selective and intercellular adhesion molecule (ICAM) [24]. The oil’s anti-inflammatory activity appears to be coupled with its ability to affect adhesion factors. Thus, spontaneous healing was promoted by avoiding the risk of ventricular rupture.

Lipophilic compounds (turmerones) were isolated from TO through hexane extraction and evaluated in a streptococcal cell wall (SCW)-induced rheumatoid arthritis murine model. Turmeric hexane extract (>28 mg/kg/day; i.p.) displayed a potent anti-inflammatory response accompanied by a high murine hepatotoxicity and mortality (56 mg/kg/day). Conversely, no toxicity signs or mortality were observed with the oral administration of this extract, employing a superior dose (560 mg/kg). This study’s authors admitted hexane extract contamination with curcuminoids [25]. Based on the preclinical and clinical evidence, the toxicity observed with TO i.p. administration may be associated with an incomplete solvent elimination or a potential synergistic effect between curcuminoids and bisabolane-type sesquiterpenes. Since it is well known that curcumin exhibits a significant oral absorption limitation [26], the absence of toxicity signs (including mortality) in using this route supports this hypothesis. In addition, extraction of TO via hydro-distillation is strongly recommended. The use of organic solvents could lead to confounding factors related to toxicity triggered by an incomplete solvent removal or unintentional collection of compounds other than those described in the regular composition of turmeric rhizome oil.

TO constitutes an exciting drug candidate for preventing, delaying, or treating cardiovascular, pulmonary, neurodegenerative, and metabolic diseases and cancer, based on the correlation of their pathogenesis to inflammation.

### 3.3. Antidiabetic

A murine model of insulin resistance evidenced the capability of TO for controlling diabetic dyslipidemia, impaired glucose tolerance, insulin resistance, and insulin sensitivity indices, and altered plasma glucose and insulin levels. The oil reduced plasma glucose (*p* < 0.05), triacylglycerides (*p* < 0.01), total cholesterol (*p* < 0.001), LDL (*p* < 0.001), malondialdehyde (*p* < 0.01); INF-δ, IL-6, and C-reactive protein levels (*p* < 0.001); and the hepatic expression of lipogenic genes (SREBP-1c, PGC-1α, and PGC-1β). Moreover, TO increased the HDL concentration (*p* < 0.001) and restored the vasorelaxation response to acetylcholine [27].

Oils obtained from fresh and dried turmeric rhizomes showed a higher glucosidase inhibitory activity than acarbose (*p* < 0.05). Ar-turmerone reduced the expression of α-glucosidase and α-amylase [28]. These results support the hypoglycemic effect of TO hexane extract containing ar-turmerone as its primary component. It was stated that ar-turmerone exerted this action through PPAR-δ activation (*p* < 0.05) [29]. Since the effect of TO from dried rhizomes was 3.5 times more than the oil from fresh samples to control α-glucosidase [28], this finding could suggest an increased concentration of ar-turmerone in dried turmeric samples.

TO’s capability for regulating SREBPAc, PGC1-α, and PGC1-β expression implies the preventive action of the oil for insulin resistance, type II diabetes, and diabetic dyslipidemia since PGC1-α promotes hepatic gluconeogenesis, PGC1-β stimulates SREBPAc expression, and SREBPAc endorses de novo hepatic lipogenesis [30,31].

### 3.4. Anticancer

The aryl hydrocarbon receptor (AhR) is a transcription factor involved in chemically induced toxicity and carcinogenesis due to its capability to generate free radicals and carcinogens. AhR has been associated with cancer, immunotoxicity, diabetes, atherosclerosis, liver fibrosis, and chronic kidney disease. A study to identify AhR ligands from the diet revealed an antagonist response of TO to human AhR probably related to the action of p-cymene [32].

Concerning oral submucous fibrosis, a precancerous oral lesion, TO prevented in vitro micronuclei formation in lymphocytes from healthy subjects. In addition, a combination of TO and turmeric extract reduced micro-nucleated cells in circulating lymphocytes and oral mucosal cells [33]. TO prevented in vitro mutagenicity caused by tobacco extract and inhibited microsomal activation of mutagens. The topical application of TO displayed anticarcinogenic activity in a murine model of skin papilloma induced by 7,12–dimethylbenz [a] anthracene and croton oil. In vitro, TO significantly hindered cytochrome p450 enzymes involved in carcinogens activation (CYP1A, A2, 2B, 2A, 2D, and 3A) (*p* < 0.001) [34]. In addition, TO prevented A431 human skin cancer cell proliferation (*p* < 0.05) and stimulated apoptosis in vitro. It is implied that these properties are related to increased caspase-3 and caspase-9 expression [35]. Activation of caspase-3, -8, and -9 mediated by TO was observed in a murine model of benign prostatic hyperplasia (BPH) [36]. Apoptosis appears to be triggered by upregulation of Bax, caspase-3, and caspase-9 and inhibition of Bcl-2 and COX-2 expression in rat tissues. TO suppressed NF-κB, a transcription factor responsible for regulating transcriptional activation of apoptosis, inflammation, cell proliferation, angiogenesis, cellular adhesion, cell invasion, and metastasis [36]. Therefore, a relevant role of NF-κB in oncogenesis promotion and cancer therapy resistance has been suggested [37]. These findings uncovered the role of NF-κB and inflammatory factors in BPH progression and turmeric oil’s contribution to treating benign hyperplasia and cancer.

Regarding cervical cancer, oral TO pre-treatment in mice with tumor xenograft implants displayed a chemopreventive effect by decreasing tumor size (*p* = 0.163). In vitro, no cytotoxic effect was observed in three cancer cell lines (HeLa, SiHa, and ME180) with a maximum concentration of 80 μg/mL [38]. Conversely, another study reported TO’s cytotoxicity against HeLa cells at higher concentrations (2100 μg/mL). TO triggered morphological changes in cancer cells and death by apoptosis [39]. TO’s chemopreventive properties may be linked to its antimutagenic action and its ability to inhibit cytochrome p450 enzymes involved in carcinogen activation [34,40]. Since ROS could act as initiators and promoters of mutagenesis and carcinogenesis, the antioxidant properties of TO may contribute to its chemopreventive action [33,41]. Nevertheless, the exact contribution of antioxidants supplementation as adjuvants in radiotherapy and certain types of chemotherapy remains unclear to date [42]. Since TO’s capability to affect the viability and morphological changes in cervical cancer cells and control tumor size corresponds to a dose-dependent relationship, it is advisable to determine the therapeutical index to better understand its antitumor activity [38,39].

A clinical study on liver cancer compared the effectiveness of hepatic arterial infusion with embolized TO vs. transcatheter artery chemoembolization (TACE). No differences were observed in the number of complete and partial remission cases, total effective rate, and incidence of post-embolism syndrome between both groups. Nevertheless, hepatic arterial infusion with TO promoted a longer survival time in liver cancer patients (*p* < 0.05) and minor myelosuppression occurrence (*p* < 0.01) than TACE treatment [43]. Additionally, TO inhibited in vitro growth of two human colon cancer cells (HT-29 and HCT-116). A murine model of HCT-116 xenograft confirmed a synergistic effect of TO with curcumin and vitamin E to inhibit cell growth in vitro and in vivo. This additive action may be attributable to the influence of TO on curcumin bioavailability, improving its absorption and distribution to the target organ [44]. The antioxidant effects of vitamin E and turmeric oil could also play an essential role in the antitumor outcome.

Interestingly, the oral administration of TO and curcumin in mice also shifted the fecal microbial composition. The *Bacteroidaceae*, *Ruminococcaceae*, *Clostridiales*, *Firmicutes*, and *Parabacteroids* families were markedly reduced, and the concentration of anti-inflammatory *Clostridium* XIVa was augmented. TO and curcumin also increased the probiotic concentration of *Lactobacillaceae* (20-fold) and *Bifidobacteriaceae* (6-fold) [45]. Additional studies are required to determine the precise association between anticancer properties and the gut microbiome composition.

### 3.5. Analgesic and Antinociceptive

An antinociceptive evaluation in mice exhibited a significant writhing reduction (*p* < 0.001) after treatment with TO. This effect was comparable to the response triggered by aspirin [19]. Accordingly, the tail-flick model evidenced the analgesic properties of TO in rats [23]. In line with these findings, the analgesic and antinociceptive properties of TO were confirmed through the hot plate and the acetic acid writhing tests. TO was capable of substantially increasing the pain threshold (*p* < 0.050) [46].

The analgesic and antinociceptive properties of TO deserve a detailed exploration. Pharmacological characterization of TO’s active ingredients is also required to further evaluate their mechanism of action and prevent possible inhibitory activities with other analgesics or related drugs. The appealing combination of analgesic and anti-inflammatory properties confers TO the status of a promissory therapeutic alternative.

### 3.6. Cardiovascular

Hemorheology assay in mice evidenced TO’s capability to decrease blood viscosity and the erythrocyte aggregation index [46]. These findings correspond to the antithrombotic activity of TO identified through the rat model of myocardial reperfusion injury. In a dose-dependent response, TO suppressed ADP-, collagen-, and thrombin-induced platelet aggregation in the presence and absence of plasma (*p* < 0.001). Thus, TO antithrombotic activity appears independent of plasma activators [8]. TO mitigated protein tyrosine phosphorylation in active platelets, augmented the total time to occlusion in arterial thrombosis induced by ferric chloride, and reduced thrombus weight (*p* < 0.001). No TO influence was identified on coagulation parameters such as prothrombin time (PT) or activated partial thromboplastin time (aPTT). The minor impact of the oil on the bleeding time and TO’s capability to diminish tyrosine phosphorylation demonstrates its potential for modulating specific pathways during platelet activation [8,27]. A thrombus-specific mechanism of action of TO with no influence on normal hemostasis is supported by the significant effect of the oil in the rat model of arterial thrombosis induced by ferric chloride, which involves platelet-rich thrombus formation. Noteworthy, TO did not exhibit cardioprotective properties in this myocardial reperfusion injury murine model. TO triggered no noticeable variation in infarct size, myeloperoxidase activity, or CK-MB serum concentrations [8]. Paradoxically to what occurs in cerebral ischemia, iNOS induction prevents cardiac injury in rats. As aforementioned, TO exerts its neuroprotective activity by reducing NOS expression, NO-mediated peroxynitrite synthesis, oxidative stress, and neuronal apoptosis. The inhibitory action of TO on NO explains the absenteeism of cardioprotective action.

Additionally, the disease-modifying capability of TO was assessed in hyperlipidemic hamsters [27,47]. TO reduced the plasma total cholesterol, LDL-cholesterol, and TAG and increased HDL-cholesterol levels. In the liver, TO decreased cholesterol synthesis and oxidative stress. TO augmented the hepatic and intestinal expression of lipid metabolism and transport genes, such as PPARa, LXRa, CYP7A1, ABCA1, ABCG5, ABCG8, and LPL. Moreover, the oil suppressed the hepatic expression of SREBP-2, HMGCR, and intestinal NPC1L1 expression.

Furthermore, TO restored eNOS mRNA expression, improved vascular function, and controlled platelet activation and oxidative stress [47]. Since lipid metabolism pathways are similar in hamsters and humans, it is suggested that the antihyperlipidemic and antiatherogenic properties of TO entangle PPARa and LXRa activation and associated enterohepatic genes involved in cholesterol absorption, transport, and metabolism [48]. PPARa, LXRa, and the target genes are responsible for cholesterol homeostasis. Lipoprotein lipase (LPL), associated with PPARa, is also activated by TO. LPL catalyzes triacylglycerol hydrolysis, thus producing antiatherogenic and hypolipidemic effects. TO also triggered LXRa and CYP7A1 activation. LXRa is responsible for the fecal excretion of cholesterol, and CYP7A1 is involved in converting cholesterol into bile acids. Accordingly, TO downregulated NPC1L1 and upregulated enterohepatic expression of ABCG5 and ABCG8 transporters, leading to a prominent decrease in cholesterol intestinal absorption [49]. ABC transporters favor the biliary excretion of cholesterol [50]. TO upregulated ABCA1 expression, thus encouraging HDL synthesis. Altogether, these mechanisms of action explain dyslipidemia improvement mediated by TO. Therefore, the cardiovascular protection exerted by TO may be a consequence of the sum of additive mechanisms of action responsible for the antioxidant, anti-inflammatory, antiplatelet, and hypolipidemic effects.

### 3.7. Neuroprotective

The pathogenesis of Parkinson’s and Alzheimer’s diseases and multiple sclerosis involves neuroinflammation. This inflammatory process is triggered by the activation of IL-6, IL-1 beta, and TNF-α. TO controlled the expression of these inflammatory cytokines in rat brains after Cd-induced neurotoxicity. In addition, the oil inhibited the activity of acetylcholinesterase (*p* < 0.01) and adenosine deaminase (*p* < 0.05). Both are key regulatory enzymes of neurodegeneration [51]. β-secretase is involved in the plaque development in the hippocampus, cerebral cortex, and amygdaloid body associated with Alzheimer’s disease pathogenesis [52]. TO inhibited in vitro β-secretase activity (53.4%), thus suggesting its potential to prevent dementia [11]. Noteworthy, turmerones, TO compounds with proven neurological activity, possess remarkable lipophilicity and brain bioavailability without showing neurotoxicity [9,11,53].

TO controlled seizure onset in zebrafish and mice models of seizures. The anticonvulsant properties of the oil and its isolated constituents were confirmed through electrophysiological analysis in zebrafish [53]. TO administration significantly decreased nitrosative stress, caspase-3 activation, and apoptosis in a murine model of cerebral infarct and corrected the mitochondrial membrane potential [54]. Findings from studies carried out in zebrafish and murine models of seizures, ischemic attack, and neuroinflammation suggest that, along with modulation of sodium ion channels, neurotransmitters signaling (γ-aminobutyric acid), and upregulation of brain-derived neurotrophic factor (BDNF), the neuroprotective properties of the oil are also related to its antioxidant and anti-inflammatory activity. The neuroprotection exerted by TO and its main components seems to be associated with its ability to up- or downregulate strategic pathways. Therefore, the antioxidant properties of turmeric oil are supported by its capability to scavenge free radicals, attenuating upregulation of Bax and Bcl-2, controlling the release of cytochrome c, inhibiting the formation of ROS and NO, and preventing lipid peroxidation and oxidative DNA damage. Additionally, TO controls neuroinflammation through the inhibition of adenosine deaminase and acetylcholinesterase. These properties guarantee the protection of tissue unaffected by neuropathogenic processes and promote a total removal of injured cells [51,53,54,55].

### 3.8. Nephroprotective

TO triggered protective activity in Cd-induced nephrotoxicity in rats and prevented modifications in renal function biomarkers (creatinine, urea, BUN), inflammatory cytokines (IL-6 and TNF-α), and renal adenosine deaminase (ADA) activity [56]. Since Cd induces a significant decrease in IL-10, an interleukin involved in cytokines synthesis inhibition, the action of TO infers a crucial role of the inflammatory response in kidney diseases.

The therapeutic application extent of TO regarding its anti-inflammatory properties must be further understood considering the crucial role of inflammation in the pathogenesis of cancer, neurological, cardiovascular, metabolic, renal, and respiratory diseases.

### 3.9. Antibacterial

TO inhibited *Porphyromonas gingivalis*, a pathogen responsible for periodontitis, showing a significant inhibition zone in vitro [57]. Likewise, TO inhibited in vitro growth, acid production, adherence to saliva-coated hydroxyapatite beads, and biofilm formation of *Streptococcus mutans*, a cariogenic bacterium [58]. *S. mutans* must adhere to tooth surfaces to metabolize dietary sugars, transforming them into lactic and formic acids. These acids lower oral pH, demineralize tooth enamel, and cause dental caries. Biofilm formation is a bacterial defense mechanism against host and antibacterial action [59]. Therefore, it is implied that TO may be helpful for the prevention and treatment of dental caries [60].

Additionally, TO restrained the growth of Gram-positive bacteria (*Bacillus cereus*, *B. coagulans*, *B. subtilis*, and *Staphylococcus aureus*) and Gram-negative bacteria (*Escherichia coli*, *Klebsiella pneumoniae*, and *Pseudomonas aeruginosa*) [61,62,63]. There is evidence that turmeric extract exerts its antibacterial activity through cell wall degradation, cytoplasmic membrane disruption, leakage of cellular components, alterations in DNA and RNA synthesis, electron transport, and nutrient uptake [64]. TO antibacterial properties are especially relevant in the current context, where antibiotic resistance is a global health problem.

### 3.10. Antifungal

Drug resistance against dermatophytes is also a growing health concern. TO showed in vitro antifungal activity against *Candida tropicalis*, *Penicillium notatum*, *Aspergillus fumigatus*, *A. niger*, *A. flavus*, *Trichophyton rubrum*, *T. violceum*, *T. mentagrophytes*, *Epidermophyton floccosum*, *Microsporum gypseum*, and *Sporothrix schenckii* [65,66,67], and a synergistic effect with the commercially available antifungal drugs: clotrimazole, fluconazole, ketoconazole, and terbinafine [65]. These findings are in line with the antifungal activity described in guinea pigs. Lesions improved after 2–5 days and completely disappeared after 6–7 days of topical TO application [66]. Ar-turmerone displayed higher anti-dermatophytic activity than ketoconazole [67]. Fungistatic and fungicidal mechanisms of the oil include structural modifications in fungal cells, functional changes, and inactivation of enzymes, proteins, and nuclear material [68]. Since no adverse skin reactions were observed in guinea pigs after topical application, TO appears to be an interesting add-on treatment for dermatological infections. Further studies focused on the underlying mechanisms of action are warranted.

On the other hand, fungal infections of cereal crops constitute a relevant health threat and economic loss factor. TO hindered mycotoxin (deoxynivalenol and zearalenone) production from *Fusarium gramineum* [69]. *F. gramineum* infection of wheat and barley causes *Fusarium* head blight, a harmful plant pathogen and mycotoxin producer. Likewise, *Aspergillus flavus* is well known for its capability to infect cereal crops and produce toxic and hepatocarcinogenic compounds. TO prevented in vitro and in vivo *A. flavus* mycelial growth, spore germination, sporulation, and aflatoxin production. Additionally, TO damaged hyphae membranes and conidiophores, controlled mycotoxin gene expression in maize, and inhibited ergosterol synthesis in fungal cells [70,71]. TO displayed in vitro antifungal activity against *Aspergillus parasiticus*, *Fusarium moniliforme*, and *Penicillium digitatum* [72]. *Fusarium verticillioides* is one of the most common producers of mycotoxins (fumonisins) in stored grains. TO moderately inhibits in vitro *F. verticillioides* growth and conidial production. A noticeable color change on *F. verticillioides* mycelium triggered by TO implies its interference in pathways related to fungal cell synthesis, affecting cell wall integrity and permeability [73]. Thus, TO appears to be a potential eco-friendly substitute for controlling fungal contamination in food since synthetic chemical fungicides produce harmful effects on crops and people.

### 3.11. Antiparasitic

TO prevented the growth of *Leishmania amazonensis* promastigotes in mice. The oil diminished the number of intracellular amastigotes and infected cells without cytotoxicity in vitro. Macrophage activation in infected mice suggests a contribution of additional mechanisms, excepting NO or ROS production [63].

Likewise, TO exhibited in vitro anti-trypanosomal activity and high selectivity indices in cytotoxicity evaluation [74]. The antipromastigote activity of TO comprises a direct action on the parasite, including structural modifications, changes in mitochondrial physiology, and cell death apoptosis. These results agree with previous reports on turmeric extract and *L. donovani* [75].

Additionally, TO displayed scolicidal properties. The oil was capable of exterminating isolated protoscoleces (in vitro) and protoscoleces into hydatid cysts collected from sheep liver (ex vivo) [76]. The major limitation in preventing protoscolex spillage during hydatid cyst surgery is the adverse effects of protoscolicide drugs (i.e., chemically induced sclerosing cholangitis) [77]. It is implied that TO exerts this activity by disrupting the protoscolex cyst wall and interfering in vital intracellular pathways. In addition, the safety evaluation of TO performed in mice showed no significant biochemical or hematological modification [76]. In this context, TO appears to be a safe and efficient antiparasitic alternative. Nevertheless, additional studies should be performed to understand this property better.

### 3.12. Insecticidal

TO displayed larvicidal activity against *Aedes albopictus*, *Aedes aegypti*, and *Culex pipiens* [78]. The oil could be used as a repellent, eco-friendly larvicide, and pupacide in breeding places. Further research on this potential application is needed in response to the current interest in substituting synthetic pesticides with natural products exhibiting improved action and minor adverse effects.

Information of published studies on the antioxidant, anti-inflammatory, antidiabetic, anticancer, analgesic, antinociceptive, cardiovascular, neuroprotective, nephroprotective, antibacterial, antifungal, antiparasitic, and insecticidal properties of TO is depicted in Table 1.

## 4. Safety

The proportion of turmeric oil constituents could vary depending on the crop’s location. Nevertheless, no toxicity warning has been reported for any of the components of the oil extracted from turmeric. Methyl eugenol, a genotoxic carcinogen, is the sole constituent of turmeric leaf oil (~3%). It is not present in turmeric rhizome oil [7,79]. Moreover, possible heavy metal contamination is negligible if the oil is extracted through steam distillation (excluding mercury) [4,80].

Reported cases of turmeric toxicity are frequently related to curcumin, a turmeric extract component that is not present in the oil [81,82,83,84]. A 13-week oral administration of TO did not cause mortality in rats or adverse effects. TO doses up to 500 mg/kg/day did not modify the hepatic and renal biochemical profiles. In addition, TO genotoxicity analysis reported no mutagenicity to Salmonella typhimurium TA98, 100, 102, and 1535. Furthermore, TO administration (1 g/kg; p.o.) for 14 days did not trigger chromosomal aberration or micronucleus formation in rat bone marrow cells [40].

Accordingly, human subjects did not show relevant adverse reactions associated with TO oral administration. Daily doses of TO (1 mL) were given to healthy volunteers for 3 months. Of nine volunteers, only two subjects discontinued the treatment: one person on the third day due to skin rash and another one on the seventh day due to intercurrent fever demanding antibiotic treatment. TO administration in the remaining seven subjects did not trigger side effects of clinical importance. Only one case of a reversible change in serum lipids was reported [85].

The evaluation of pharmacological properties and toxicity is relevant in searching for novel, efficient, and safe therapeutic alternatives. In the case of TO, no undesired effects have been described in animals or humans. In addition, carcinogens are not present in the oil composition [40,43,85]. Consistent with these findings, even computational models for carcinogenicity prediction pointed to ar-turmerone, a major TO constituent, as a non-mutagenic, non-carcinogenic, and non-hepatotoxic agent with negligible side effects [84]. These characteristics support the classification of TO as ‘Generally Recognized As Safe (GRAS)’ conferred by the FDA [40].

## 5. Conclusions

Based on the available information, the bioactivities of the oil are not modified by oral, intravenous, or intraperitoneal administration. The dose–response relationship observed with the oil and its role as a bioavailability enhancer suggest an appropriate absorption and distribution. Furthermore, the high brain bioavailability of the oil supports its therapeutic application for various illnesses, including neurological diseases, due to its ability to overcome the blood–brain barrier.

The therapeutic potential of TO deserves scientific attention due to the vast diversity of possible pharmacological targets. These include antioxidant, anti-inflammatory, analgesic, antinociceptive, neuroprotective, cardiovascular, antidiabetic, nephroprotective, anticancer, antibacterial, antifungal, antiparasitic, and insecticidal properties. Most research studies described in this review were carried out at the preclinical level, reporting interesting pharmacological effects without associated toxicity. Since TO’s safety was confirmed in healthy volunteers, the development of clinical research on TO’s active compounds remains a pending matter.

TO is a rich source of bioactive molecules. Major chemical constituents are often pointed out as the responsible compounds for the oil’s pharmacological properties. Nevertheless, the proportion of each component in the oil can be affected by the crops’ geographical location, plant nutritional status, or maturity stage. Thus, the reported bioactivities of TO should be considered a baseline for the development of further research on the isolated compounds since the oil’s activity will not always depend on its primary constituents. Due to its potency, a minor component could be accountable for a specific response. Moreover, the oil′s pharmacological effect could result from the joint action of two or more compounds leading to an enhanced, synergistic, or inhibitory outcome.

Further studies are required to assess the potential clinical application of TO’s active constituents. It remains crucial to determine the pharmacological profile of the isolated TO active compounds and their bioavailability, efficacy, and safety to maximize their therapeutical benefits according to the target organ.

## Figures and Tables

**Table 1:** A summary of studies focused on the bioactivities of turmeric oil.

Bioactivity	Main Compounds of Turmeric Oil	Model	Concentration/Dose; Administration Route	Source
**Antioxidant**	ar-turmerone, a-turmerone, β-turmerone	In vitro	0.025 g/3 mL	[17]
	ar-turmerone, β-turmerone, ar-curcumene	In vitro	80% ethanol	[18]
	ar-turmerone, curlone, ar-curcumene	In vitro	200 mg/mL	[19]
		In vivo (mouse)	250 mg/kg; i.p.	[19]
	ar-turmerone, a-turmerone, β-turmerone, curlone	In vivo (rat)	250 mg/kg; i.p.	[20]
**Anti-inflammatory**	ar-turmerone, curlone, ar-curcumene	In vivo (mouse)	500 mg/kg; i.p.	[19]
	ar-turmerone, a-turmerone, β-turmerone	In vivo (rat)	100 mg/kg; p.o.	[23]
	ar-turmerone, turmerone, and curlone	In vivo (rat)	250 mg/kg; p.o.	[24]
	ar-turmerone, α-turmerone, β-turmerone	In vivo (rat)	>28 mg/kg/day; i.p.	[25]
		In vivo (rat)	560 mg/kg; p.o.	[25]
**Antidiabetic**	ar-turmerone, a-turmerone, β-turmerone, curlone	In vivo (hamster)	300 mg/kg/day; p.o.	[27]
		In vivo (rat)	300 mg/kg/day; p.o.	[27]
	ar-turmerone	In vitro	0.38 mg/mL	[28]
	ar-turmerone	In vivo (mouse)	0.5 g/100 g of diet; p.o.	[29]
**Anticancer**	p-cymene	In vitro	100 μg/mL	[32]
	not reported	In vivo (human)	600 mg TO + 3 g turmeric extract; p.o.	[33]
	ar-turmerone	In vitro	1 mg/plate	[34]
		In vivo (mice)	50%; topical	[34]
		In vitro	200 mg/mL	[34]
	not reported	In vitro	80 mg/L	[35]
	not reported	In vitro	40 mg/mL	[36]
		In vivo (rat)	7.2 mg/kg; i.g.	[36]
	ar-turmerone, a-turmerone, β-turmerone	In vitro	2100 mg/mL	[39]
	ar-turmerone, curlone	In vitro	3000 mg/plate	[40]
		In vivo (rat)	1 g/kg; p.o.	[40]
	not reported	In vivo (human)	3 mL (embolized)	[43]
	ar-turmerone, a-turmerone, β-turmerone, a-santalene, ar-curcumene	In vivo (mice)	5 mg (TO + curcumin)/kg; p.o.	[45]
**Analgesic and antinociceptive**	ar-turmerone, curlone, ar-curcumene	In vivo (mouse)	100 mg/kg; i.p.	[19]
	ar-turmerone, a-turmerone, β-turmerone	In vivo (rat)	100 mg/kg; p.o.	[23]
	ar-turmerone, curlone, turmerone	In vivo (mouse)	9.75 mL/kg; i.p.	[46]
**Cardiovascular**	ar-turmerone, a-turmerone, β-turmerone, curlone	In vivo (rat)	1 g/kg; p.o.	[8]
		In vivo (mouse)	1 g/kg; p.o.	[8]
	ar-turmerone, a-turmerone, β-turmerone, curlone	In vivo (hamster)	300 mg/kg/day; p.o.	[27]
		In vivo (rat)	300 mg/kg/day; p.o.	[27]
	ar-turmerone, curlone, turmerone	In vivo (mouse)	9.75 mL/kg; i.p.	[46]
	ar-turmerone, a-turmerone, β-turmerone, curlone	In vivo (hamster)	300 mg/kg; p.o.	[47]
**Neuroprotective**	ar-turmerone, a-turmerone, β-turmerone, l-zingiberene, β-sesquiphellandrene	In vitro	250 mg/mL	[11]
	eucalyptol	In vivo (rat)	50 mg/kg; p.o.	[51]
	ar-turmerone, a-turmerone, β-turmerone, a-atlantone	In vivo (zebrafish)	10 mg/mL	[53]
		In vivo (mouse)	100 mg/kg; i.v.	[53]
	ar-turmerone, a-turmerone, β-turmerone, curlone	In vivo (rat)	50 mg/kg, p.o.	[54]
**Nephroprotective**	eucalyptol	In vivo (rat)	50 mg/kg; p.o.	[56]
**Antibacterial**	not reported	In vitro	100%	[57]
	a-turmerone, germacrone	In vitro	>0.5 mg/mL	[58]
	not reported	In vitro	1000 ppm	[61]
	ar-turmerone, turmerone, curlone	In vitro	100 ppm	[62]
	turmerone, b-turmerone, γ-curcumene	In vitro	75 mL	[63]
**Antifungal**	z-citral	In vitro	10 mg/mL	[65]
	not reported	In vitro	114.9 mg/mL	[66]
		In vivo (guinea pig)	topical	[66]
	ar-turmerone	In vitro	6% *w*/*w*; topical	[67]
	not reported	In vitro	11,580 mg/mL	[69]
	ar-turmerone, turmerone, b-sesquiphellandrene, curcumene	In vitro	4 mL/mL	[70]
	ar-turmerone, a-turmerone, β-turmerone	In vitro	0.5% *v*/*v*	[71]
	ar-turmerone, a-zingiberene, b-(Z)-farnesene, ar-curcumene	In vitro	6 mg/mL	[72]
	ar-turmerone, a-turmerone, β-turmerone	In vitro	1000 ppm	[73]
**Antiparasitic**	turmerone, β-turmerone, γ-curcumene	In vitro	500 mg/mL	[63]
	a-zingiberene, b-sesquiphellandrene, ar-turmerone, curlone	In vitro	3.17 nL/mL	[74]
	a-turmerone, b-turmerone	In vitro	200 mg/mL	[76]
**Insecticidal**	turmerone, curcumene	In vivo (mosquito larvae)	0.2 mg/mL	[78]

## Data Availability

Not applicable.

## Abbreviations

ABC	adenosine triphosphate-binding cassette
ADA	adenosine deaminase
AhR	aryl hydrocarbon receptor
aPTT	activated partial thromboplastin time
Bax	B-cell leukaemia/lymphoma 2-associated X Protein
Bcl	B-cell lymphoma 2
BDNF	brain-derived neurotrophic factor
BPH	benign prostatic hyperplasia
CK-MB	creatine kinase MB
COX	cyclooxygenase
CYP	cytochrome P-450
DHA	docosahexanoic acid
eNOS	endothelial NOS
EPA	eicosapentaenoic acid
GRAS	Generally Recognized As Safe
HMGCR	hydroxymethylglutaryl coenzyme A reductase
ICAM	intercellular adhesion molecule
i.g.	intragastric
IL	interleukin
iNOS	inducible nitric oxide synthase
i.p.	intraperitoneal
i.v.	intravenous
LXR	liver X receptor
LPL	lipoprotein lipase
MI/RP	myocardial ischemia/reperfusion
NF	nuclear factor
NO	nitric oxide
NOS	nitric oxide synthase
NPC1L1	Niemann-Pick C1-Like 1
PGC	peroxisome proliferator-activated receptor gamma coactivator
p.o.	per os (oral)
PPAR	peroxisome proliferator-activated receptors
PT	prothrombin time
ROS	reactive oxygen species
SCW	streptococcal cell wall
SREBP	sterol regulatory element binding protein
TACE	transcatheter artery chemoembolization
TAG	triacylglycerides
TNF	tumor necrosis factor
TO	turmeric oil
